# Claudin2 is involved in the interaction between *Megalocytivirus*-induced virus-mock basement membrane (VMBM) and lymphatic endothelial cells

**DOI:** 10.1186/s13567-024-01404-9

**Published:** 2024-11-06

**Authors:** Jian-hui He, Deyu Han, Xianyu Meng, Lingling Li, Bangping Hu, Muting Yan, Zi-Ang Wang, Shaoping Weng, Jianguo He, Xiaopeng Xu

**Affiliations:** 1 State Key Laboratory of Biocontrol, School of Life Sciences/ Southern Marine Science and Engineering Guangdong Laboratory (Zhuhai)/ China-ASEAN Belt and Road Joint Laboratory On Mariculture Technology, Sun Yat-Sen University, Guangzhou, China; 2 Institute of Aquatic Economic Animals and Guangdong Province Key Laboratory for Aquatic Economic Animals, Sun Yat-Sen University, Guangzhou, China

**Keywords:** *Megalocytivirus*, virus-mock basement membrane, pathogenesis, Claudin

## Abstract

The genus *Megalocytivirus*, belonging to the family *Iridoviridae*, is one of the most detrimental virus groups to fish aquaculture. *Megalocytivirus* creates a virus-mock basement membrane (VMBM) on the surface of infected cells. This membrane provides attachment sites for lymphatic endothelial cells (LECs), disrupting fish's endothelial cell-extracellular matrix system. This disruption triggers injury to the vascular system and can result in death. Exploring the VMBM-cell interaction mechanism is crucial for uncovering the pathogenesis of *Megalocytivirus* and identifying therapeutic targets. Claudins, a class of tetra transmembrane proteins, play a key role in creating tight junctions between endothelial or epithelial cells. In this study, we demonstrated that the expression of Claudin2, a member of the Claudin family in fish, was significantly up-regulated by *Megalocytivirus* infection. Claudin2 was found in LECs attached to the surface of infected cells. It interacted with the VMBM viral components VP23R, VP08R, and VP33L at multiple binding sites through its two extracellular loops. However, it did not interact with the host basement membrane’s nidogen. Therefore, Claudin2 is involved in the interaction of LEC with VMBM and plays a role in the disturbed distribution of extracellular matrix and endothelial cells in *Megalocytivirus*-infected fish tissues. This study aims to uncover the molecular mechanisms by which *Megalocytivirus* infection leads to pathological changes in the vascular system.

## Introduction

The family *Iridoviridae* is a group of icosahedral cytoplasmic DNA viruses with large circularly permuted and terminally redundant DNA genomes [1]. The genus *Megalocytivirus* represents a set of iridoviruses that infect fish, causing enlargement of infected cells in almost all tissues, particularly the spleen and kidney [2]. *Megalocytivirus* infection can lead to 100% mortality, causing significant economic losses in the fish farming industry. Infectious spleen and kidney necrosis virus (ISKNV) is the type species of the genus *Megalocytivirus* and infects more than 60 fishes, including mandarin fish *Siniperca chuatsi*, zebrafish *Danio rerio*, green-spotted pufferfish *Tetraodon nigroviridis*, and largemouth bass *Micropterus salmoides* [3–7].

Fish infected with megalocytiviruses display symptoms such as anoxia, hemorrhage in the body surface and internal organs, pale gills, anaemia, and sometimes ascites [8, 9]. This is all suggestive of damage to the vascular system. The basic structure of vascular tubes is formed by the basement membrane (BM), which is lined with blood endothelial cells (BEC) or lymphatic endothelial cells (LEC) [10, 11]. This indicates that *Megalocytivirus* pathogenicity may be associated with pathological endothelial cells (ECs) and extracellular matrix (ECM) changes. Interestingly, studies on ISKNV have shown that the enlarged infected cells in tissues are covered by a layer of flat cells with flat nuclei. These cells are positive for the LEC-specific markers Prox-1, VEGFR-3, and LYVE-1 and can be identified as LECs [12, 13]. Electron microscopy revealed a low electron-density structure about 50 nm thick, which is half the thickness of the BM, between the infected cells and attached LECs. This unique structure, called the virus-mock basement membrane (VMBM), imitates the functions of a real BM and provides attachment sites for LECs. The VMBM comprises the viral proteins VP23R, VP08R, and VP33L, which imitate the functions of the BM components laminin and collagen IV, forming a sandwich-like structure [12–14]. The nidogen protein rivets the laminin framework and collagen IV layers in the real BM [15]. It is also involved in VMBM formation by interacting with VP23R [13]. The viral components of VMBM can control the growth, movement, and tube formation of LECs via the VEGFR-3 signalling pathway, possibly related to the origin of LECs adhering to the surface of infected cells [12].

LEC adherence to the surface of infected cells via VMBM is a characteristic feature of the vascular system’s pathological changes caused by *Megalocytivirus* infection. Unravelling the mechanism of the functional association between VMBM and LEC is important for revealing the virulence mechanism of *Megalocytivirus* and potentially provides referential clues for studying vasculogenesis. A recent study revealed that the interaction of VMBM components with the LEC-specific markers VEGFR-3 and LYVE-1 contributes to the selective adhesion of LECs on VMBM [12]. However, the molecular basis of the VMBM-LEC interaction remains largely unknown, limiting the exploration of the pathogenesis of *Megalocytivirus* infection.

Claudins, a family of membrane proteins containing four transmembrane domains with the N-terminus and the C-terminus in the cytoplasm, are widely expressed in endothelial and epithelial cells and are essentially involved in the formation of the epithelial/endothelial tight junctions [16–18]. Through their extracellular domains, claudin proteins on the membranes of two adjacent cells interact to establish a paracellular barrier controlling molecule flow across the intercellular space [19–21]. The current study suggested that a claudin from *S. chuatsi*, expressed in the attached LECs, was involved in the interaction between VMBMs and LECs. This finding may contribute to further investigations into the function of VMBMs and the pathogenic mechanism of *Megalocytivirus*.

## Materials and methods

### Animals and virus

The mandarin fish used in this study were the same as those in a recent study with ethical approval of animal use protocol from the Institutional Animal Care and Use Committee of Sun Yat-sen University (SYSU-IACUC-2021-B1245) [12]. The ISKNV stocks were produced in MFF-1 cells cultured in DMEM medium containing 10% foetal bovine serum at 27 °C, and the virus titres were determined by a 50% tissue culture infective dose (TCID_50_) assay [22].

### qPCR

The cDNA was prepared from *S. chuatsi* spleen tissues collected 1–5 days after ISKNV infection, as previously described [23]. They were used as a template in a 10 μL PCR amplification system containing 5 μL 2 × SYBR Premix Ex TaqTM II (Takara, Japan) and 500 nM of each primer. The qPCR reaction was performed with parameters of 95 ℃ for 30 s followed by 40 cycles of 95 ℃ for 15 s, 60 ℃ for 15 s, and 72 ℃ for 10 s on a LightCycle 480 System (Roche, Germany). The primers C1-F/C1-R (5′-ATGGTGGAATGGAAAGCCTC-3′/5′-TGCCCTGTACAATCCCTGGT-3′), C2-F/C2-R (5′-GACCTTCTACCGACCCAACG-3′/5′-GTCTCCCACGACCACCATCC-3′), C3-F/C3-R (5′-CGGCCAACACCATAATCAGG-3′/5′-GAGCAGTCCGCCACCAAGAA-3′), and 18S-F/18S-R (5′-AAGACGGACGAAAGCGAAA-3′/5′-GGCGGGTCATGGGAATAAC-3′) were used for analysing *S. chuatsi* Claudin1 (Genbank accession No. XM_044220397.1), Claudin2 (XM_044223465.1), Claudin3 (XM_044203082.1) genes and the internal control 18S rRNA, respectively. Three independent experiments were conducted, each with three parallel real-time PCR tests, and similar results were obtained.

### Immunofluorescence

To analyse the expression and localisation of Claudin2 in vivo, immunofluorescence assays were performed on sections of *S. chuatsi* spleen tissues at 0, 1, 2, 3, 4, and 5 days post ISKNV infection [12]. After deparaffinization, rehydration, and antigen-repairing, sections were blocked with 10% normal goat serum. They were then incubated with a rabbit antibody against Claudin2 (customised from GL Biochem, China) and a mouse antibody against MCP or VP23R [13]. They were subsequently developed using Alexa Fluor 488-conjugated goat anti-rabbit IgG antibody (Cat#ab150077, Abcam, UK) and Alexa Fluor 594-conjugated secondary anti-mouse antibody (Cat#ab150120, Abcam, UK). Sections were then nuclear-stained with Hoechst 33258 (Sigma-Aldrich, USA) and visualised on a Leica LSM 410 confocal microscope (Germany) at 488 and 596 nm excitation wavelengths.

### Co-immunoprecipitation (Co-IP)

The coding sequences of Flag- or GFP-tagged full lengths or fragments of ISKNV VP33L, VP08R, VP23R, and *S. chuatsi* nidogen-1 and Claudin2 were cloned into pcDNA3.1A vector (Invitrogen, USA) and co-transfected into HEK293T cells as previously described [12]. Cells were lysed 48 h post-transfection and subjected to Co-IP and reciprocal Co-IP assays using anti-Flag Magnetic Beads (Cat#HY-K0207, MedChemExpress, USA) and anti-GFP Agarose Affinity Gel (Cat#D153-8, MBL, Japan) respectively. The input cell lysates and precipitated proteins were analysed by western blot using anti-Flag (Cat#14793, CST, USA) and anti-GFP (Cat#SAB4301138, Sigma-Aldrich, USA) antibodies. The in vivo interaction was analysed in lysed spleen tissues of ISKNV-infected *S. chuatsi* at 5 dpi using Protein A/G plus agarose (Cat#sc-2003, Santa Cruz, USA) and the rabbit antibody against *S. chuatsi* Claudin2 (customised from GL Biochem China). The precipitated samples were analysed by western blot using previously reported antibodies against VP23R and VP08R [12].

### Pulldown

The coding sequences of GFP (as control) and GFP-tagged Claudin2 were cloned into the pcDNA3.1A vector (Invitrogen, USA) and transfected into MFF-1 cells. After 48 h, the cells were infected with ISKNV at a multiplicity of infection (MOI) of 3. They were then harvested for lysis at 72 h post-infection. The lysates were incubated with anti-GFP Agarose Affinity Gel (Cat#D153-8, MBL, Japan) for 30 min at 4 °C. The gel elution was analysed by western blot using anti-VP33L antibody.

## Results

### Expression of Claudin2 in LECs attaching on ISKNV-infected cells

**Figure 1:**
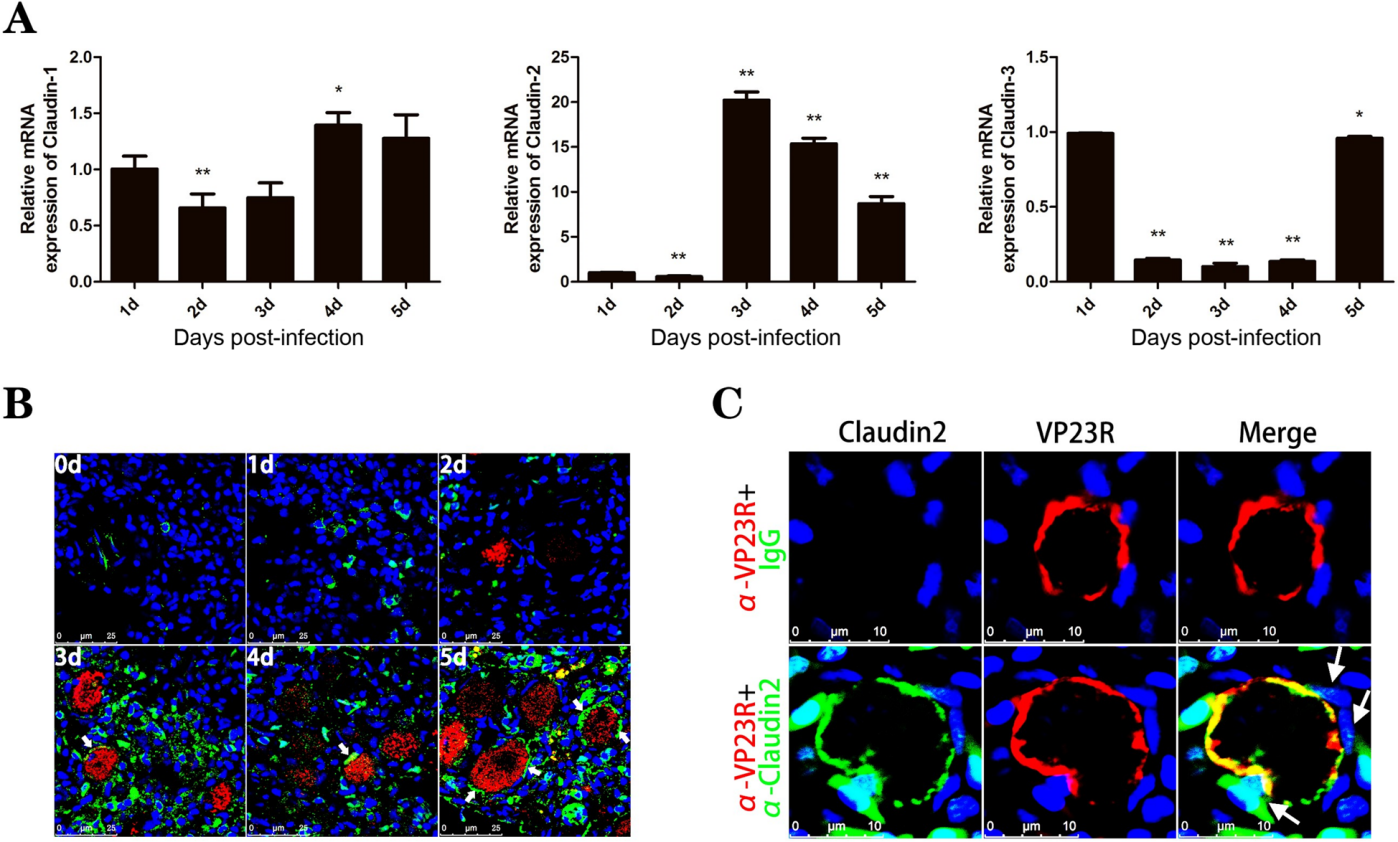
**Expression and localisation of Claudin2 in ISKNV-infected tissues.** **A** Expression of three Claudin genes in the spleen from ISKNV-infected *S. chuatsi* analysed by qPCR. The data shown are representative of three experiments and presented as means ± SD of three detections. *p < 0.05, **p < 0.01 by one-way ANOVA with Dunnett's post hoc test. **B** Immunofluorescence analysis of Claudin2 (green) in spleen tissues of *S. chuatsi* at 0, 1-, 2-, 3-, 4-, and 5-days post-ISKNV infection. The cytoplasm and the nucleus were marked by anti-MCP antibody (red) and Hoechst 33,258 (blue). **C** Claudin2 (green) was present on the surface of ISKNV-infected cells marked by anti-VP23R antibody (red) in *S. chuatsi* spleen tissues at 5 dpi. White arrows indicate the fluorescence signals of Claudin2 in LECs attaching on infected cells.

The expression profiles of three Claudin family members of *S. chuatsi* after ISKNV infection were investigated using qPCR (Figure 1A). The results showed that in the spleen tissues, the mRNA level of claudin1 underwent a slight change from 1 to 5 d after ISKNV infection, while that of Claudin3 was down-regulated from 2 to 4 days post-infection (dpi). In contrast, the expression of Claudin2 increased significantly on day 3 dpi to 20.2-fold that of 1 dpi and was maintained at 15.4-fold and 8.7-fold levels on 4 and 5 dpi, respectively. This result was consistent with the immunofluorescence assays, which showed a significant increase in the fluorescent signals of Claudin2 protein in spleen tissues after 3 dpi (Figure 1B). The signals of Claudin2 were poorly distributed in ISKNV-infected tissues on 1 dpi. However, after 3 dpi, they were widely present in the gaps between infected cells and appeared in sheets on the surface of infected cells. These cells contained intracellular virions marked by the major capsid protein (MCP) [24]. Magnified views showed that the signals of Claudin2 overlapped with those of VP23R, both displaying a sheet-like distribution on infected cell surfaces (Figure 1C). These findings suggest that Claudin2 is localised to LECs that adhere to the surface of cells infected with ISKNV.

### The interaction between Claudin2 and VP08R

**Figure 2:**
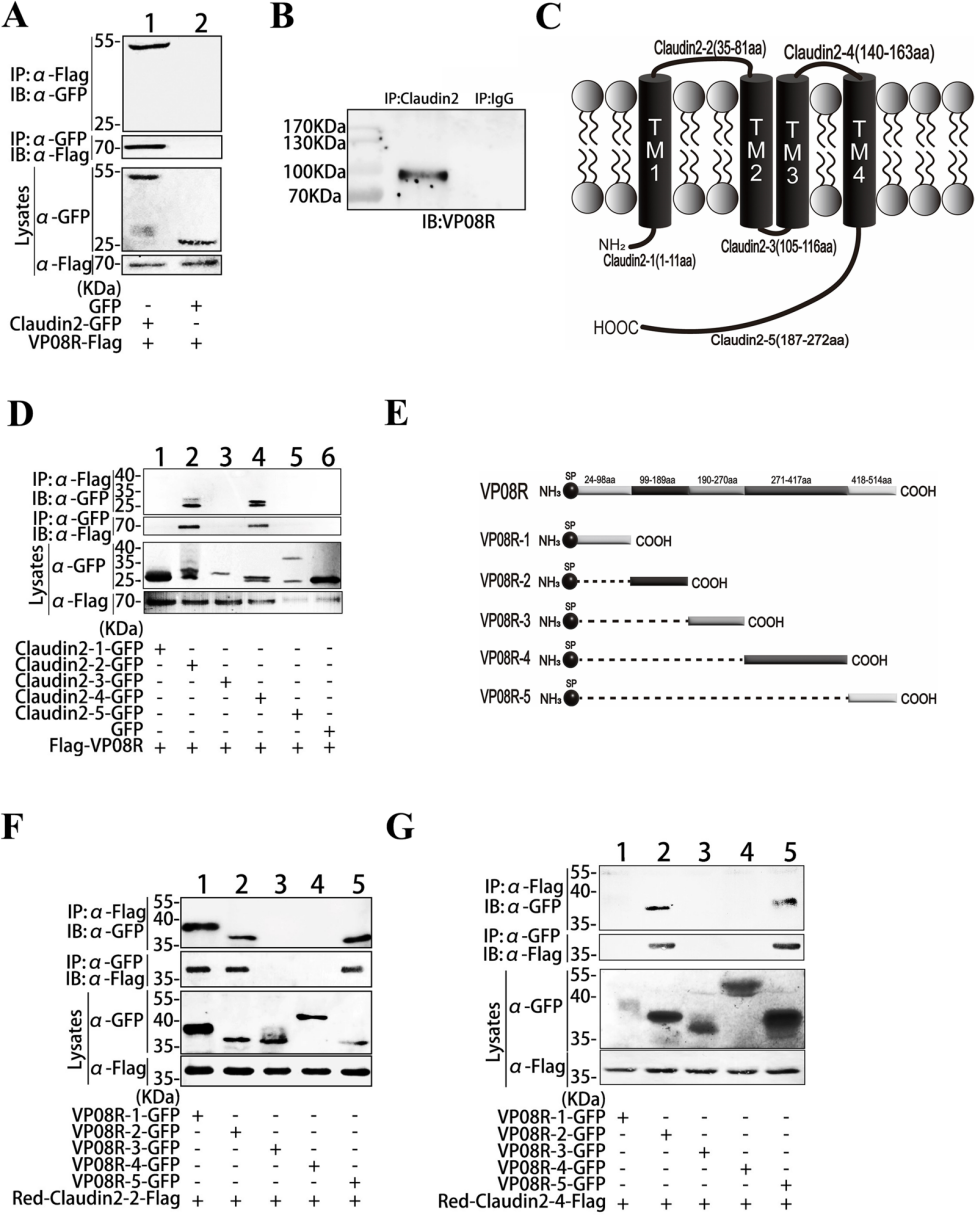
**Interaction of Claudin2 with VP08R.** **A** Co-IP and reciprocal Co-IP analysed the interaction between Claudin2 and VP08R in transfected HEK293T cells. **B** Co-IP analysed the interaction between Claudin2 and VP08R in ISKNV-infected spleen tissues. **C** Diagram of the Claudin2 fragments. **D** The interaction of VP08R with the fragments of Claudin2 analysed by Co-IP. **E** Diagram of the VP08R fragments. **F, G** The interactions of VP08R fragments with the 35–81 aa (**F**) and 140–163 aa (**G**) regions of Claudin2 analysed by Co-IP.

VP08R forms multimers in VMBM to mimic the role of Collagen IV in the real BM [14]. To investigate whether Claudin2 is involved in the VMBM-LEC association, the interaction between Claudin2 and VP08R was analysed. Co-IP assays using co-transfected HEK293T cells indicated that the GFP-tagged Claudin2 interacts with the Flag-tagged VP08R, confirmed by reciprocal Co-IP (Figure 2A). The interaction between endogenous Claudin2 and VP08R in ISKNV-infected spleen tissues was further determined by Co-IP and western blot using their specific antibodies (Figure 2B). Next, the fragments of the non-transmembrane regions of Claudin2 were segmentally expressed (Figure 2C), then subjected to Co-IP analysis to examine the binding sites of Claudin2 to VP08R. The results showed that the first and the second extracellular loop regions (35–81 aa and 140–163 aa) of Claudin2 interacted with VP08R (Figure 2D). The screening analyses of VP08R fragments found that the 35–81 aa region of Claudin2 (Claudin2-2) can bind to the 24–189 aa and 418–514 aa regions of VP08R. Additionally, it was observed that the 140–163 aa region of Claudin2 (Claudin2-4) interacted with the 99–189 aa and 418–514 aa regions of VP08R (Figure 2E–G).

### The interaction of Claudin2 with VP23R

**Figure 3:**
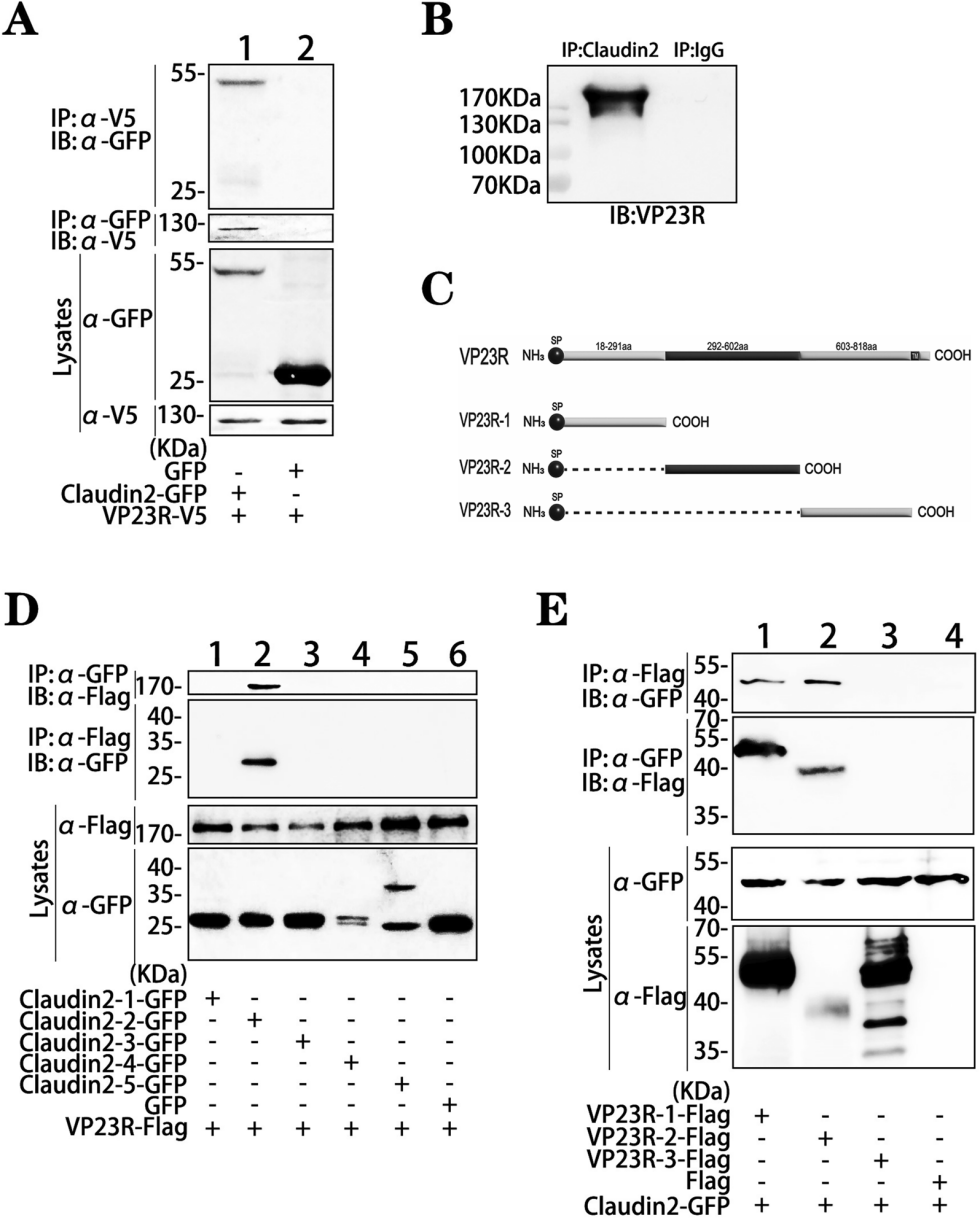
**Interaction of Claudin2 with VP23R.** **A** Co-IP and reciprocal Co-IP analyses of the interaction between Claudin2 and VP23R in vitro. **B** Co-IP analysis of the interaction between Claudin2 and VP23R in vivo. **C** Diagram of the VP23R fragments. **D** Co-IP analysis of the interaction of VP23R with the fragments of Claudin2. **E** Co-IP analysis of the interaction of VP23R fragments with the 35–81 aa region of Claudin2.

VP23R contains a C-terminal transmembrane domain and an extracellular region similar to laminin γ1. This region interacts with the host BM component nidogen to initiate the assembly of VMBM, hijacking the membrane of infected cells and mimicking the laminin network of real BM in VMBM [13]. The in vitro Co-IP and reciprocal Co-IP experiments revealed that Claudin2 could interact with VP23R in transfected HEK293T cells (Figure 3A). In vivo Co-IP analysis confirmed the interaction between ISKNV-encoded VP23R and endogenous Claudin2 in *S. chuatsi* spleen tissues (Figure 3B). Further analysis in vitro revealed that the first extracellular region of Claudin2 binds to VP23R (Figure 3D), while the N-terminus region (18–602 aa) of VP23R is responsible for this interaction (Figure 3C, E).

### The interaction of Claudin2 with VP33L and nidogen

**Figure 4:**
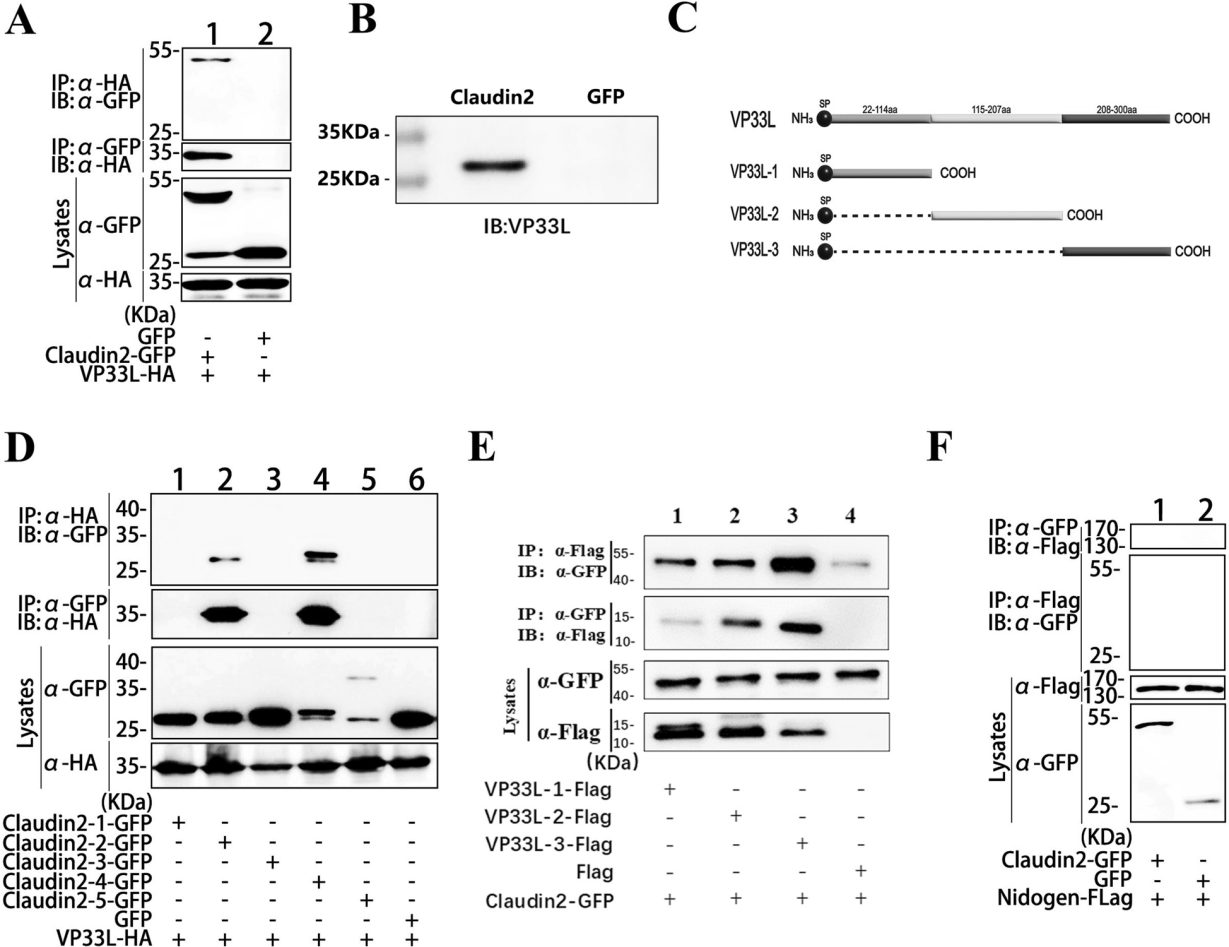
**Interaction between Claudin2 and VP33L.** **A** Co-IP and reciprocal Co-IP analyses of the Claudin2-VP33L interaction. **B** Pulldown assay of the interaction between GFP-tagged Claudin2 and ISKNV-encoded VP33L in MFF-1 cells. **C** Diagram of the VP33L fragments. **D** Co-IP analysis of the interaction of VP33L with Claudin2 fragments. **E** Co-IP analysis of the interaction of Claudin2 with VP33L fragments. **F** No interaction was observed between Claudin2 and nidogen.

VP33L is a secreted protein encoded by ISKNV which has recently been identified as a novel component of VMBM [12]. VP33L is located on the surface of ISKNV-infected cells and is involved in the assembly of VMBM by interacting with VP08R but not VP23R. Co-IP assessed the binding of VP33L to Claudin2 and reciprocal Co-IP in HEK293T cells (Figure 4A). In further pulldown assays, it was demonstrated that GFP-tagged Claudin2 could precipitate the VP33L protein expressed by ISKNV in *S. chuatsi* fry cells (MFF-1 cells) (Figure 4B). The interaction between VP33L and Claudin2 was observed in all the analysed VP33L fragments (Figure 4D) and in the two extracellular regions of Claudin2 (Figure 4C, E). Among the three fragments of VP33L, the C-terminal region (208–300 aa) interacts most strongly with Claudin2, while the N-terminal region, 22–114 aa, has the weakest interaction.

**Figure 5:**
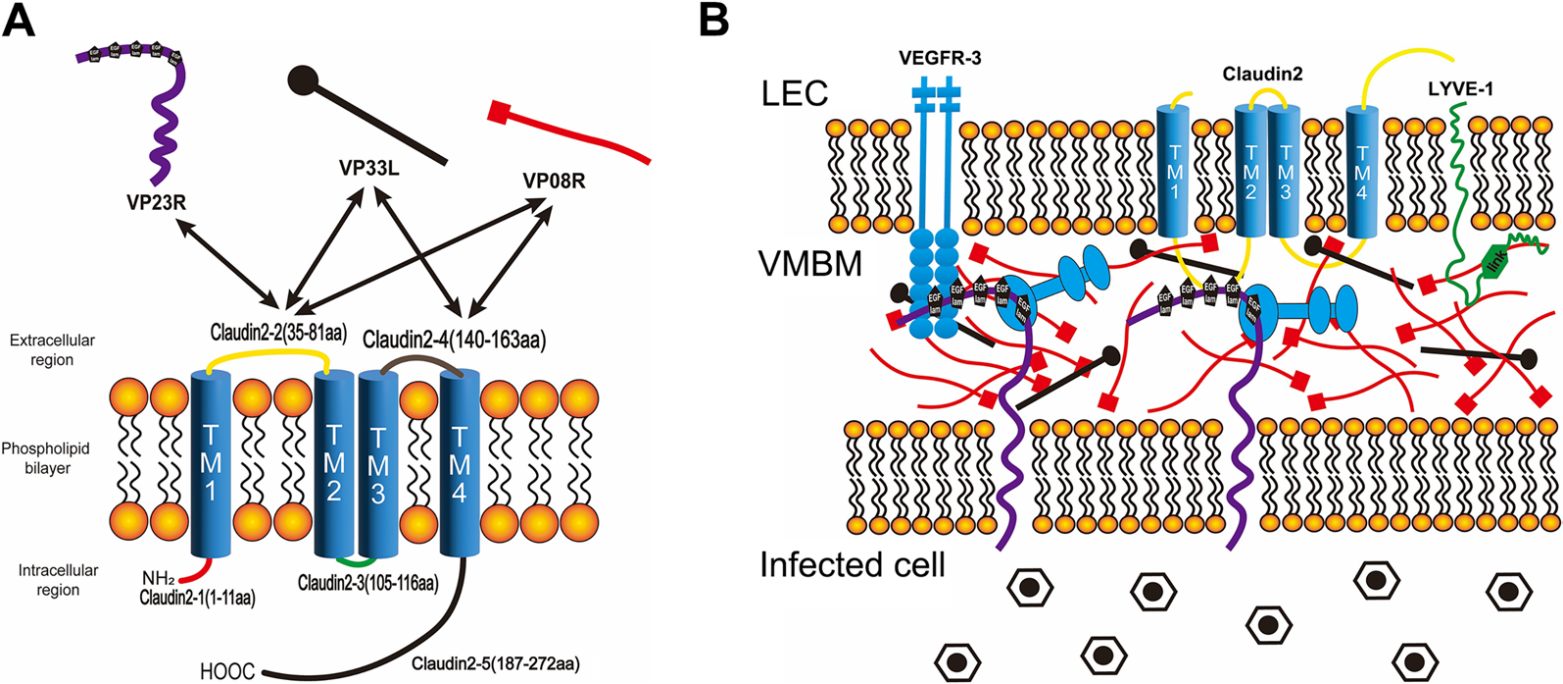
**Diagram of the interaction between Claudin2 and VMBM components.** **A** Interactions between the extracellular loop regions of Claudin2 and VMBM components. Two-way arrows indicate the interaction between each other. **B** The structure of VMBM and its interaction with LECs. In addition to the proteins labelled in the diagram, the blue dumbbell-shaped structure indicates the nidogen protein.

The above results suggest that Claudin2 could interact with all the analysed ISKNV-encoded components of VMBM (diagrammatically shown in Figure 5A). However, there was no interaction between Claudin2 and nidogen (Figure 4F). This study and previous research further refined the structure of VMBM and its interaction mechanism with LECs (Figure 5B).

## Discussion

Claudins are typically located within the junction complex between adjacent endothelial or epithelial cells in normal tissues. They prohibit the passage of molecules and ions through the paracellular space [25, 26]. Some claudins can regulate cell–matrix adhesion by affecting the expression of integrins and matrix metalloproteinases [27–29]. However, there has been little focus on the direct interaction of claudins with ECMs. This study demonstrated that claudin was involved in the interaction between a virus-mock BM-like structure and endothelial cells in fish, providing clues to the study of Claudin family interactions with ECMs. The qPCR analysis in this study showed that ISKNV infection selectively increases the mRNA levels of Claudin2 in *S. chuatsi* tissues. Immunofluorescence assays have shown an increase in the number of cells expressing Claudin2 molecules with the duration of infection. Considering that Claudins are mainly expressed in epithelial or endothelial cells, this implies that ISKNV infection alters the behaviour of endothelial cells. This could also be a mechanism contributing to the pathological changes in the vascular system, which warrants further in-depth study.

The BM has a sandwich structure, consisting of a laminin polymer layer, a collagen IV network, and the nidogen protein acting as a rivet molecule to cross-link the two layers [30–32]. Laminin plays a central role in the self-assembly of BMs and mediates the attachment of cells to BMs by binding to specific cellular receptors [33–35]. As a *Megalocytivirus*-mimicked BM-like structure with a streamlined form, VMBM has a distinct interaction with cells. The membrane protein VP23R mimics the γ1 chain of laminin. In the actual BM, this chain extends out of the laminin layer to bind and recruit nidogen. As a result, the plasma membrane of infected cells takes on the role of the laminin polymer layer [13]. VMBM lacks the collagen IV and is only half the thickness of real BM. Instead, a secreted viral protein VP08R (~ 90 kDa) forms cross-linked multimers through intermolecular disulfide bonds. This protein interacts with VP23R and nidogen-1, imitating the functions of the collagen IV network to uphold the integrity and stability of VMBM [14]. The expression of VP08R was regulated by a viral microRNA, suggesting that the formation and maintenance of VMBM could be finely controlled by ISKNV [36]. The recently identified viral VP33L protein indirectly interacts with VP23R by binding to VP08R, giving a sandwich-like structure to VMBM [12]. The current study demonstrated that all three major viral components of VMBM interact with the attached LECs on the surface of ISKNV-infected cells by interacting with Claudin2. The extracellular region of Claudin2 is short, but it can interact with all known VMBM viral components. The first extracellular loop of Claudin2 is 46 aa long and can interact with VP23R, VP08R, and VP33L. The second extracellular loop of Claudin2 is only 24 aa long but can interact with both VP08R and VP33L. The VMBM components interact with Claudin2 at multiple sites. For example, the binding sites for the Claudin2-2 fragment (the first extracellular loop) on VP08R are present on the three analysed VP08R fragments, and those for the Claudin2-4 fragment (the second extracellular loop) are present on two fragments. This suggests that the structure of VMBM allows all of its viral components to make contact with the surface of the attached LEC.

The BM, lined with vascular endothelial cells or LECs, forms the fundamental structure of blood vessels or lymphatic vessels, respectively [10]. However, BMs’ composition and basic architecture in blood vessels and lymphatic vessels are identical. There are no reports on the mechanisms by which the BM in these two types of vascular tubes interacts with different types of endothelial cells. In contrast, in ISKNV-infected tissues, VMBM specifically provided attachment sites for LECs but not vascular endothelial cells. A recent study revealed that membrane proteins VEGFR-3 and LYVE-1, which are specific markers for LECs, play a role in the interaction between VMBM and LECs [12]. Specifically, VP23R, VP08R, and VP33L can bind to VEGFR-3, while VP08R can interact with LYVE-1. This interaction may be responsible for the specific attachment of LECs to VMBM. This study demonstrated that *S. chuatsi* Claudin2 is expressed not only in LECs adhering to the VMBM, but also in other tissue cells. This suggests that Claudin2 is unlikely to be a LEC-specific molecule. The interaction of Claudin2 with VP23R, VP08R, and VP33L through its outer-membrane regions may enhance the adhesion of LECs to the VMBM.

The only host protein identified to be involved in VMBM composition is nidogen [13]. Previous studies have demonstrated that nidogen is also involved in cell adhesion to the BM by binding to cellular receptors [37–39]. However, although nidogen is involved in the assembly of VMBM by interacting with VP23R, it is not involved in the interaction of VMBM with VEGFR-3 and LYVE-1. This study also showed that nidogen did not interact with Claudin2. The role of nidogen in the interaction between VMBM and LEC requires further investigation. Moreover, VMBM components can regulate the proliferation and migration of LECs, which could partially explain the origin of LECs on the surface of ISKNV-infected cells. It has been reported that Claudins can also transmit extracellular signals to regulate the behaviour of cells [40–42]. Whether the interaction with VMBM components can also affect LEC activity deserves further investigation.

## Data Availability

All data generated or analysed during this study are included in this published article.
